# Oncological and Functional Outcomes After Type III Cordectomy for Early Glottic Cancer (Tis, T1a): A Retrospective Study Based on Our 10-Year Experience

**DOI:** 10.3390/jcm13237164

**Published:** 2024-11-26

**Authors:** Eleonora Lovati, Elisabetta Genovese, Livio Presutti, Marco Trebbi, Luca Pingani, Gian Maria Galeazzi, Maria Pia Luppi, Matteo Alicandri-Ciufelli, Daniele Marchioni, Maria Consolazione Guarnaccia

**Affiliations:** 1 Otorhinolaryngology-Head and Neck Surgery, Department of Diagnostic Clinical and Public Health, University of Modena and Reggio Emilia, 41125 Modena, Italy; elisabetta.genovese@unimore.it (E.G.); matteo.alicandriciufelli@unimore.it (M.A.-C.); daniele.marchioni@unimore.it (D.M.); guarnaccia.maria@aou.mo.it (M.C.G.); 2 Department of Medical and Surgical Sciences (DIMEC), Alma Mater Studiorum, Università di Bologna, 40138 Bologna, Italy; 3 Department of Otolaryngology—Head and Neck Surgery, IRCCS Azienda Ospedaliero-Universitaria di Bologna, 40138 Bologna, Italy; 4 Otolaryngology Head and Neck Surgery Department, Ospedale Infermi di Rimini, 47923 Rimini, Italy; 5 Department of Biomedical, Metabolic, and Neural Sciences, Università degli Studi di Modena e Reggio Emilia, 41125 Modena, Italygianmaria.galeazzi@unimore.it (G.M.G.)

**Keywords:** early glottic cancer, larynx, cordectomy, trans-oral laser microsurgery, oncology

## Abstract

**Background:** The recommended treatment for early glottic cancer is trans-oral laser microsurgery, with excellent oncological and functional outcomes. The aim of this study is to evaluate oncological and functional outcomes in patients who underwent monolateral type III laser cordectomy for early glottic cancer. **Methods:** A total of 104 patients were enrolled. Staging, histological type, grading, assessment of surgical margins, mean time of relapse, OS, DFS, and DSS were obtained. Maximum phonation time, GIRBAS score, shimmer, jitter, fundamental frequency, and Yanagihara score were evaluated. Patients were submitted to the VHI-10 questionnaire. **Results:** Correlations between patients with single recurrence and the anterior commissure involvement were analyzed, as well as correlations between patients with recurrence and the status of margins. Correlations between VHI-10 scores and anterior commissure involvement were analyzed. **Conclusions:** The recurrence rate was higher in patients with anterior commissure involvement. A significant inversely proportional association between DSS and assessment of surgical margins was observed. The distribution of VHI-10 scores differed significatively in patients with and without anterior commissure involvement. Vocal results reflected mild dysphonia.

## 1. Introduction

Larynx cancer is the second-most frequent cancer of the head and neck district. It is more frequent in males in the 4–5 decades of life and in elderly patients, and it is associated with smoking, abusing alcohol, and inhalation of irritant agents. Other conditions, such as reflux disease, HPV infection, and laryngeal papilloma, are comorbidities that may be associated with the etiology of this type of cancer [1,2].

In glottic cancer, one or both vocal folds are involved, with a possible extension to the anterior commissure. Therapeutic strategies contemplate open neck surgery, trans-oral laser microsurgery (TLM), or external beam radiotherapy [1,2]. TLM is nowadays the recommended treatment for early glottic cancer (Tis, T1a, T1b, T2), with excellent oncological outcomes reported in the literature since the 1970s, reporting high rates of overall survival (OS), disease-free survival (DFS), and disease-specific survival (DSS) [2,3,4,5,6,7,8,9,10,11,12,13,14,15]. Lee et al. [8] showed a rate of OS of 92.2%, a rate of DFS of 87.9%, and a rate of DSS of 99%. Lester et al. [9] reported values of OS of 88.8%, DFS of 96.2%, and DSS of 98.1%. Authors such as Chiesa et al. [10], Lucioni et al. [11], and Del Mundo et al. [13] reported rates of OS, DFS, and DSS greater than 90% in their patients, with high rates of laryngeal preservation.

Early glottis cancer is an intraepithelial lesion that does not go over the basement membrane. T1 tumors are limited to vocal cords, which have normal mobility; these lesions are divided between T1a, involving one vocal cord, and T1b for the involvement of both vocal cords. T2 tumors extend to supraglottis or subglottis, with compromised vocal cord motility [16].

In 2000, the European Laryngological Society (ELS) published a classification of endoscopic cordectomies [2,15,17], according to the extension of cancer and the type of resection required, in order to create a common classification useful to compare outcomes between different centers [6]. There are 9 types of transoral laser cordectomy according to ELS [15,17]. Transmuscular cordectomy (type III) consists of a laser resection through the vocal muscle that could extend from the vocal process to the anterior commissure. During this procedure, partial resection of the ventricular fold is required to expose the vocal fold. This technique is recommended for small superficial cancers (Tis, T1a, T1b, T2) that do not deeply infiltrate the vocal muscle.

In the evaluation of patients subjected to TLM, surgical margin status and the involvement of anterior commissure should be considered, since patients with positive margins are recommended to have a close laryngostroboscopy follow-up and since patients with involvement of anterior commissure have a higher risk of recurrence [18,19].

It is important to consider the functional outcome in terms of voice quality after this type of intervention because it is correlated to quality of life [10,14,20]. The quality of voice depends on the type of cordectomy performed and on the extension of the primary lesion, which contribute to defining the phonatory compensation after surgical treatment. In several studies, laser cordectomies are reported to have good functional results, preserving an acceptable phonatory function and a good glottal closure, rather than open neck surgery and radiation therapy—even if there are controversial results [20,21,22,23]. In the literature are reported variations in fundamental frequency, in maximum phonation time (MPT), in the GIRBAS scale (grade, instability, roughness, breathiness, asthenia, and strain) and in parameters such as Jitter and Shimmer as a consequence of TLM [20,21,22,23]

The aim of this retrospective monocentric study is to evaluate the oncological and functional outcomes after laser monolateral type III cordectomy for early glottic cancer in a 1-year to 10-year follow-up.

## 2. Materials and Methods

In this retrospective study, 132 patients subjected to monolateral laser type III cordectomy for Tis and T1a glottic cancer between 2010 and 2020, who attended the Otorhinolaryngology Department of the University Hospital of Modena, were enrolled. Patients with type I cordectomy or vocal fold biopsy as a part of their diagnostic pathway before type III cordectomy were included.

Patients were subjected to this procedure after a biopsy positive for cancer limited to one vocal fold, since their clinical evaluation with video-laryngostroboscopy (XION Medical, Berlin, Germany) was highly suspicious for a malignant lesion.

The 28 patients who were not available to analyze oncological and voice outcomes after surgical treatment were excluded, thus analyzing 104 patients.

Overall survival, disease-specific survival and disease-free survival, staging, histological type, grading, and assessment of surgical margins were evaluated according to the histopathological findings. The mean time between tumor and its relapse, first and second relapse, first tumor and contralateral tumor, contralateral tumor and its relapse was calculated.

Periodic video-laryngostroboscopy was performed during follow-up.

For functional analysis, the following steps were performed:

- The maximum phonation time (MPT) was first evaluated, distributing the patients in 4 subgroups based on MPT duration. The first group included patients with MPT values higher than 10 s—the normal value; the second group patients with MPT slightly reduced (8–10 s); the third group patients with MPT moderately reduced (5–7 s); and the fourth group patients with MPT severely reduced (<5 s).
- The GIRBAS score (grade, instability, roughness, breathiness, asthenia, and strain) was obtained with a team of expert speech therapists, collating results in a score from 0 to 3 and dividing them in 4 groups: normal (0), slightly impaired (1), moderately impaired (2), and severe impaired (3) parameters. These results were acquired recording a voice message (name, surname, vowels, numbers from 1 to 10, and a sung verse of a well-known Italian song) with a 20 cm distant microphone positioned in front of the patient at the level of the mouth, connected with the software LingWaves 3 Voice Clinic Suite Pro on a computer.
- Parameters such as shimmer, jitter, fundamental frequency of vowel /a/, and fundamental frequency of the word /aiuole/ were calculated using LingWaves software; only male patients were considered for the analysis of F0 because only 1 female patient was submitted to the analysis.
- Yanagihara score was assessed on the spectrogram of the word /aiuole/, collocating the results in a score from 1 to 4 (normal, slightly impaired, moderately impaired, severely impaired), dividing patients in 4 groups according to the result obtained.
- The singing voice phonetogram of the vowel /a/ was analyzed with both normal and whispering voice, from the lowest frequency to the highest frequency according to the ability of the patient and the surgical outcomes.
- The maximum and minimum intensity in dB and the maximum and minimum frequency were achieved, observing the result of the phonetogram. Then, the range in decibels—achieved from the difference between maximum and minimum intensity—and the range in semitones—obtained from the difference between maximum and minimum frequency—were calculated with a logarithmic formula. The normal value for the semitones range is greater than 10 semitones; for the decibel range, it is greater than 22 dB. The maximum intensity of the fundamental frequency of the word /aiuole/ was found in order to obtain the maximum intensity of speech voice.
- Finally, 34 patients were submitted to the Voice Handicap Index-10 questionnaire (VHI-10) to evaluate the subjective perception of voice. This questionnaire has 10 items, with 5 answer options from “0” (never) to “4” (always). Patients were divided into 4 subgroups based on the score obtained from the compilation of VHI-10: in the first group we collocated patients with a score of 0 (normal score), in the second group between 1 and 13 (slightly improved), in the third group from 14 to 27 (moderately improved), and in the fourth group from 28 to 40 (severely improved).

The GIRBAS scale was analyzed in patients who were subjected to speech therapy and in patients who were not treated, correlating these values with VHI-10 questionnaire results and with the involvement of anterior commissure.

Parameters such as jitter, shimmer, F0 of the vowel /a/, and the word /aiuole/ were examined in patients subjected to speech therapy, comparing them with those who were not treated after surgery and analyzing the correlations between these scores, the results of the VHI-10 questionnaire, and the involvement of anterior commissure.

Yanagihara’s score results were reported in patients who undertake speech therapy and in those who do not, correlating them to the involvement of anterior commissure.

Finally, the results of the VHI-10 questionnaire were compared between patients who undertake speech therapy and those who do not, and between patients with anterior commissure involvement and without.

The study conforms to the 1976 Declaration of Helsinki and has been approved by the coordinating center ethics committee (Policlinico di Modena University Hospital) (Protocol n.0011684/22—ID-3735).

The statistical analysis was performed by an expert statistician, in particular using chi-square analysis and Kendall’s Tau and Spearman’s rank correlation coefficient. The Kaplan–Meier survival function was applied to assess the DFS, DSS, and OS rates. A *p* < 0.0001 was considered significant.

## 3. Results

Among all patients, 96% were male and 4% female. The mean age at diagnosis was 67 years (ranging from 28 to 90 years). A total of 22 patients (21%) underwent surgery extended to anterior commissure. Only 34 patients underwent speech therapy.

Oncological results have been calculated on the 104 patients who were periodically examined during the time of follow-up, analyzing overall survival (OS), disease-specific survival (DSS), and disease-free survival (DFS).

Periodic video-laryngostroboscopy examinations of 76 patients were performed in the first year every three months, in the second and third years every six months, and in the fourth and fifth years every 10–12 months. The other 28 patients were periodically examined in other Italian Otolaryngology centers.

Functional results have been calculated on 49 patients who voluntarily submitted to spectro-acoustic analysis of voice after TLM, 34 of which were submitted to the Voice Handicap Index-10 questionnaire and 26 of which underwent speech therapy.

### 3.1. Oncological Results

Data from 104 patients subjected to MLS type III cordectomy were collected. The mean age at diagnosis is 67 years. Forty-seven patients had a diagnosis of in situ carcinoma (45%) with a stage 0 disease; fifty-seven had a T1a cancer (55%) with a stage I disease. The histological type of tumors was squamous cell cancer (95%), sarcomatoid carcinoma (1%), leiomyosarcoma (1%) and high-grade dysplasia (3%). In 14.4% of cases, the tumor was well-differentiated (grade 1), in 20.2%, it was moderately differentiated (grade 2), and poorly differentiated in 4.8% (grade 3). The grade of the remaining patients has not been reported in the histological examinations. For the assessment of surgical margins, seventy-seven patients (74%) had negative margins at the histopathological evaluation, seventeen patients (16.4%) had close resection margins, and ten (9.6%) had positive margins (Table 1).

In the group on positive and close margins, three patients relapsed. The mean time between first diagnosis and tumor recurrence is 3.36 years; the mean time between first and second relapse is 1.68 years. The mean time between the diagnosis of the first and the contralateral tumor is 2.2 years; the mean time between the contralateral tumor and its recurrence is 0.88 years. Eleven patients had disease relapse during follow-up (11%), three of whom had a second relapse (2.9%). Eleven patients had contralateral vocal fold cancer during follow-up (11%), two of whom had a relapse (1.9%) (Table 2).

Eight relapsed patients were submitted to revision of the previous surgery, undergoing cordectomy on the same vocal fold previously resected. Two of those patients had a second relapse and underwent total laryngectomy. One relapsed patient was submitted to total laryngectomy because of old age and comorbidities associated. Two patients, after a second cordectomy for a contralateral tumor, had a relapse of the first cancer and underwent open partial horizontal laryngectomy type IIA (OPHL2A) (Table 3).

**Figure 1:**
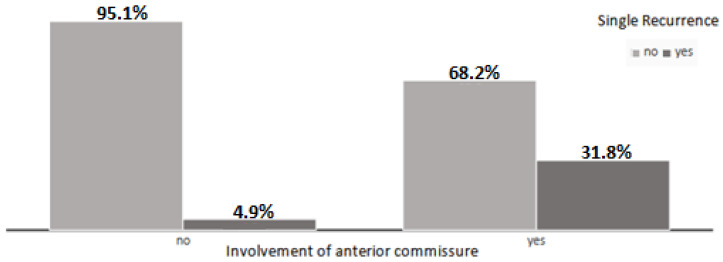
Differences in distribution of patients with a single recurrence and the distribution of patients who underwent extended resection to anterior commissure.

The mean follow-up was 4.8 years. Over the 10 years, 45 patients completed 5 years of follow-up. Only two patients (2%) underwent radiotherapy after surgery. Eighteen patients died during follow-up (17%), but, in only one case, it was disease-related. No patient reported dysphagia. Among patients with more than 5 years of follow-up (43%), the 5-year disease-free survival was 93%, the disease-specific survival was 100%, and the overall survival was 95%. For patients with a less than 5-year follow-up (57%), DFS was 93%, DSS was 98%, and OS was 75%. Using chi-square analysis, the distribution of patients with a single recurrence and the distribution of patients who underwent extended resection to anterior commissure were noticed to differ significantly, since 31.8% of patients with extended resection relapsed, compared to 4.9% of relapsed patients submitted to simple cordectomy (x^2^ = 13.31; d.f. = 1; *p* < 0.0001) (Figure 1).

Any significant difference was observed between the distribution of patients submitted to simple cordectomy without any recurrence or second tumor and the distribution of those with contralateral tumor, such as between patients with contralateral tumor and those who underwent cordectomy extended to the anterior commissure.

The percentage of patients with disease recurrence did not differ significantly between groups of patients divided according to the status of surgical margins.

Finally, there is a significant negative association between DSS and the assessment of surgical margins, so with close and positive margins, DSS inversely proportional decreases (Tau = −0.24; *p* = 0.003; Rho = −0.288; *p* = 0.003).

### 3.2. Functional Results

Data from 49 patients submitted to type III cordectomy were collected, according to the SIFEL procedure. The functional results were correlated with VHI-10 responses, with the involvement of the anterior commissure, and with the execution of speech therapy treatment.

#### 3.2.1. Maximum Phonation Time Analysis

For the MPT, the results of 42 patients were analyzed. The mean value was 11.15 s. Patients were divided into four groups, as described in the previous paragraph. Twenty-four patients (57%) showed excellent performance with a normal MPT (<10 s); three patients (7%) had a slight reduction in the MPT; eight patients (19%) had a moderate reduction in the MPT; and finally, seven patients (17%) had a severe reduction in the MPT. In the first group, 10 out of 24 patients (41.7%) underwent speech therapy treatment; in the second group, 1 out of 3 patients (34%); in the third group, 6 out of 8 patients (75%); and in the last group, 4 out of 7 patients (57%). Analyzing patients who were subjected to speech therapy, 10 out of 34 patients (29.4%) had normal MPT.

#### 3.2.2. GIRBAS Scale

**Figure 2:**
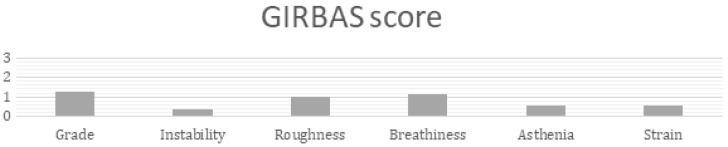
Mean values of GIRBAS parameters.

Data from the GIRBAS scale were collected on 49 patients, placing them in four subgroups (Figure 2). Results of GIRBAS score are shown in Table 4.

Using chi-square analysis, the distribution of patients with different scores of grade, instability, breathiness, and asthenia did not differ significantly between who underwent speech therapy and who did not (respectively, x^2^ = 4.47, d.f. = 3, *p*: 0.215; x^2^ = 1.18, d.f. = 2, *p*: 0.554; x^2^ = 3.81, d.f. = 3, *p*: 0.283; x^2^ = 1.77, d.f. = 3, *p*: 0.622). From the chi-square analysis, the distribution of patients with different scores of roughness and strain differs significantly between who underwent speech therapy and who did not (respectively x^2^ = 13.31; d.f. = 1; *p* < 0.0001) (Table 5).

Analyzing the distribution of patients in the 4 scores and the involvement of anterior commissure, no significant differences were observed between patients with involvement of anterior commissure and the grade of alteration of GIRBAS parameters. The mean of GIRBAS parameters was 0.82, configuring mild perceptive dysphonia.

#### 3.2.3. Jitter, Shimmer, and Fundamental Frequency

Values of acoustic parameters such as shimmer, jitter, and fundamental frequency (F0) of the vowel /a/ were obtained using LingWaves software. Results of 49 patients were analyzed. The mean jitter value was 2.18%, and the mean shimmer value was 13.84%. For the analysis of fundamental frequency (F0) of the word /aiuole/, only male patients (n = 48) were considered because only one female patient was submitted to acoustic voice analysis. The mean F0 value was found to be 170.55 Hz. The normal range of F0 was from 80 Hz to 150 Hz. 14 patients (29.17%) collocated in the range of normality.

Using chi-square analysis, no statistically significant differences were observed between the distribution of the values of these parameters in patients treated by a speech therapist and the ones not subjected to rehabilitative treatment. In other words, the distribution of these values was the same for the two groups of patients (subjected and not to speech therapy). Furthermore, no statistically significant differences were noticed in the distribution of the values of these parameters in patients with and without anterior commissure involvement.

#### 3.2.4. Yanagihara Score

The Yanagihara score was evaluated on 49 patients, analyzing the spectrogram of the word /aiuole/ and then collocating them in four subgroups. Seven patients (14.29%) had normal results, fifteen (30.61%) had a slight alteration of the spectrogram, twenty-four (48.98%) had a moderately altered spectrogram, and three (6.12%) had a severe alteration.

Using chi-square analysis, no statistically significant differences were observed in the distribution of Yanagihara score values in patients treated by a speech therapist and patients not subjected to rehabilitative treatment, and with or without anterior commissure involvement.

#### 3.2.5. Singing Voice Phonetogram

The singing voice phonetogram was analyzed in 48 patients. The range in decibel, achieved from the difference between maximum and minimum intensity, was normal in 47.9% of patients (n = 23) and altered in 52.1% of patients (n = 25).

The range in semitones, obtained from the difference between maximum and minimum frequency with a logarithmic formula, was normal in 47.9% of patients (n = 23) and altered in 52.1% of patients (n = 25).

The maximum intensity of the fundamental frequency of the word /aiuole/ in the phonetogram ranges between 72 dB and 99 dB, with a mean value of 85.35 dB.

#### 3.2.6. Voice Handicap Index-10

Only 37 patients were submitted voluntarily to the VHI-10 questionnaire. Nine patients (24.32%) had normal results, twenty-four (64.87%) had a slight alteration of the spectrogram, four (10.81%) had a moderately altered spectrogram, and no patients had a severe alteration. The mean value collocated in the slight alteration. No correlations were observed between jitter (Tau = 0.216; *p* = 0.124 and Rho = 0.258; *p* = 0.141), shimmer (Tau = 0.094; *p* = 0.503 and Rho = 0.121; *p* = 0.495), and F0 (Tau = 0.058; *p* = 0.677 and Rho = 0.73; *p* = 0.680) values of vowel /a/ and the results of the VHI-10 questionnaire.

No correlations were found between F0 values of the word /aiuole/ and the results of the VHI-10 questionnaire (Tau = −0.058 *p* = 0.677 and Rho = −0.066; *p* = 0.712).

There was no evidence of correlations between the grade of alteration of the GIRBAS scale and the results of the VHI-10.

Using chi-square analysis, no statistically significant differences were observed in the distribution of VHI-10 scores in patients submitted or not to speech therapy.

With chi-square analysis, the distribution of the scores obtained at the VHI-10 questionnaire showed to differ in a statistically significant way between patients with and without anterior commissure involvement (x^2^ = 15.41; d.f. = 2; *p* < 0.0001).

## 4. Discussion

### 4.1. Oncological Outcomes

Advantages of TLM are the study of margins with the possibility of a second approach in case of positive margins and the possibility of reuse of this technique in case of recurrence. Disadvantages are the risk of a residual glottic air gap after the procedure, the anesthetic risk, and a specialized center with surgical materials [14,18].

In this study, TLM has been demonstrated to be a safe procedure with no associated mortality and very low complications, since no patients died after the surgical procedure; the only patient who died of the disease had a recurrence treated with total laryngectomy (TL) and had several comorbidities. No patient had postoperative dysphagia, as noticed by different authors [1,18,19,24]. TL is a surgical procedure used for advanced laryngeal cancers, but it can be performed as a salvage treatment for cancer recurrence after an organ-sparing treatment such as TLM, as reported in one patient in this study. This procedure needs a structured postoperative rehabilitation program made by speech therapists in order to improve patients’ quality of life. Vocal performances can be obtained by learning esophageal speech or by the insertion of a tracheoesophageal prosthesis [25]. The most common early complication of TL is the pharyngocutaneous fistula, whose risk is higher if surgery is performed after radiotherapy [25,26,27]. Reconstructive techniques such as the use of flaps are usually performed in order to prevent complications and improve quality of life [27]. In 2010, Groselj and Fajdiga [28] described epiglottoplasty as an option for pharyngeal surgical reconstruction with a good locoregional control rate and satisfying functional outcomes for patients’ voices [25,28].

In line with the literature, TLM could be repeated with a larger resection in case of recurrence, reserving radiotherapy for second primary tumors or second recurrences. Furthermore, open laryngectomy can be performed after TLM in cases of recurrence requiring a more extended resection [13,14,24].

The 5-year DSF and DSS values reported in this study are in accordance with those reported in the literature, with a 5-year DSF over 85% and a DSS over 90% [18,19,24,29].

In this study, the percentage of recurrence was higher in patients who underwent TLM extended to anterior commissure (31.9%) than in patients subjected to simple cordectomy (4.9%), as noticed by many authors [18,19,30]. However, results are controversial since some authors observed no differences in recurrence and survival between patients with T1a cancer with or without involvement of anterior commissure [24,31]. TLM is recommended for tumors extended to the anterior commissure since radiotherapy has a lower disease control rate [19], but it could be a difficult procedure if the exposure is not adequate [24,32].

No significant differences were observed between the group of patients submitted to simple cordectomy and those with contralateral tumors, such as between patients with contralateral tumor and those who underwent cordectomy extended to the anterior commissure. It could be hypothesized that the involvement of commissure and having a vocal fold tumor are not associated with having a contralateral tumor.

A total of 27 patients (26%) were found to have close and positive resection margins, 3 of which relapsed, but the percentage of recurrence did not differ significantly between patients with or without positive margins, as previously described by Del Mundo A. et al. [14].

The rate of positive margins was 27% in patients with anterior commissure involvement and 26% in patients without involvement on commissure, in contrast to what was reported by Chone et al. [19]. In the case of positive margins, a close laryngostroboscopy follow-up is required to exclude the recurrence of the disease, since the literature describes a relation between positive margins and a higher rate of local recurrence [19].

An interesting inverse relation between DSS and surgical margin status was observed, but further studies are required to assess this correlation. The other main treatment for early glottic cancer is radical radiotherapy (RT). This option is available in many centers, and it is a safe procedure with good oncological outcomes with good loco-regional control and survival. Its advantages are that it could be performed in patients without good exposure to the larynx, preserving more tissues. However, RT has some advantages, such as tissue swallowing and actinic edema, glottis stenosis, xerostomia, the impossibility of reusing this technique, and difficulties in performing further surgery—in case of recurrence, it is possible to perform a total salvage laryngectomy [33,34]. Comparing these two options in the literature, oncological results are similar with both procedures. In the case of TLM, Guimaraes et al. [33] reported that DSS was greater in patients treated with TLM than RT, with a lower risk of performing a total laryngectomy in case of relapse.

### 4.2. Functional Outcomes

In the literature, functional outcomes are controversial, and voice quality parameters have not been standardized yet. For example, there is a lot of software with different cut-offs and different parameters [14]. Many authors show that the grade of dysphonia increases with the resection to a greater extent [14,22,29,35,36] since TLM causes diminution of vocal fold vibration and may generate an incomplete glottal closure [14,29].

The involvement of anterior commissure is usually associated with the impairment of the compensation mechanisms, causing poor results in terms of voice quality. This is due to a larger glottic gap, a more extended resection, and the presence of an anterior glottis web [22,23,29,37,38,39]. However, there are controversial results about the involvement of the anterior commissure, since some authors observed that vocal results depend maybe on the depth of the resection instead of the extension [40,41]. In contrast with what was reported by many authors [22,23,29,38,39], in this study, no significant differences were noticed between the involvement of anterior commissure, the grade of alteration of GIRBAS values, the distribution of F0, jitter, and shimmer values, and the Yanagihara score. Voice quality perception (examined with the VHI-10 questionnaire) was worse in patients submitted to resection extended to the anterior commissure, as reported by Rho et al. [37].

VHI-10 is an important tool to evaluate the quality of life of patients subjected to TLM since it reflects the impact of voice disorder perception and management on the quality of life. The patients were submitted to speech therapy after surgical resection, though in the literature there are very few works on vocal results after therapy, and it could be difficult to compare the results. The purpose of speech therapy is to support glottic compensation and avoid supraglottic hypercontraction [42].

Analyzing results about vocal parameters, in the literature MPT values post-MLS are controversial; however, some authors reported an improvement of mean values 12 months after surgery. [14,39] Lee et al. [22] showed no significant differences with the preoperative values, whereas Hartl et al. [23] and Sjogren et al. [36] observed a slight impairment of the MPT mean value, mostly associated with a larger resection. However, in the literature, the majority of MPT mean values reported are in the range of normality (>10 s), and our findings are in line with these results [20,22,29,36,39,42].

Speech therapy can improve MPT after surgery. The results of this work demonstrate that patients with poor MPT needed to be subjected to speech therapy, since without therapy their performances would have been worse than the ones reported.

In the literature, many authors evaluated the GIRBAS score and reported a mild grade of dysphonia after MLS [20,23,29,32,33,36]. Lee et al. [22] observed in their study an immediate deterioration of these parameters with a subsequent improvement after 6 months, comparing the last results to the preoperative condition; they also noticed an improvement in the “G” and “R” scores when the anterior commissure was not involved. The results of this study are in line with the literature, showing a mean value indicative of mild perceptive dysphonia. Furthermore, the few patients with moderate to severe altered scores are more represented in the group who did speech therapy, as to indicate the extreme need for these patients to undertake a rehabilitative program.

The results about fundamental frequency, Jitter%, and Shimmer% reflected mean values reported in the literature [22,29,42,43]. Lee et al. [22] and Peretti et al. [44] did not notice significant differences between preoperative and postoperative values, whereas Lechien et al. [42] showed an improvement of both Jitter% and Shimmer% 12 months after surgery and Del Mundo et al. [14] only of Shimmer% values. Chu et al. [39] reported good results in cases of limited resections.

In the literature, several authors try to compare voice outcomes between patients submitted to TLM and to irradiation treatment. In their study, Mehel et al. [45] did not find significant differences in VHI-10 and GRBAS score, as well as Mendenhall et al. [46], who observed similar vocal results in patients submitted to TLM and radiotherapy for T1a glottic cancers. Greulich et al. [47] did not find a significant difference in VHI after TLM and RT. Yılmaz et al. [48] did not notice any significant differences between the two groups, whereas Aaltonen et al. [49] noticed only a difference in breathiness, which was better after irradiation treatment than after TLM. Our results are in line with what is reported by these works and reflect that TLM can give similar voice outcomes to patients, suggesting that treatment option choice for early glottis cancer should be evaluated considering factors like age, comorbidities, performance status, cost of the procedure, occupation, and patient preference. However, results are controversial since some authors noticed better vocal outcomes in patients submitted to RT. Tomifuji et al. [50] reported in their study similar outcomes for type I and II cordectomy and RT, whereas RT had a better outcome compared to type III cordectomy, probably due to the extension of the resection. Krengli et al. [51] observed that voice quality was better after radiotherapy than after cordectomy.

The limits of this study are its retrospective design, the monocentric setting, the small number of data, the patients lost at follow-up, a second TLM, which could confound the vocal results, and a subsequent laryngectomy, which does not permit to evaluate vocal outcomes. Further studies are required to establish the association between commissure involvement and margin status and to investigate the correlation between margin status and survival. Furthermore, a standardization of protocols of voice analysis is mandatory to compare the data, and more studies on the involvement of anterior commissure are required to establish the grade of impairment of vocal parameters. The establishment of a standardized protocol for voice analysis may be useful to compare patients from different centers submitted to the same surgical procedure. With this study, the SIFEL protocol is recommended as a standardized method to assess the severity of post-surgical dysphonia.

## 5. Conclusions

TLM is a safe surgical procedure with no associated mortality and very low complications. It can be compared with RT, with the advantage of MLS being repeated in case of recurrence or a second primary tumor. In this study, 5-year DSS and DFS are similar to those reported in the literature.

The percentage of recurrence is higher in patients with anterior commissure involvement. Having a second primary tumor is not associated with recurrence of the primary tumor or involvement of anterior commissure. Recurrence does not seem to be correlated to surgical margin status.

Voice outcomes show mild dysphonia. The greater the extent of the resection, the more impaired the functional outcome. Results about the involvement of the anterior commissure are controversial.

## Figures and Tables

**Table 1:** Percentages and numbers of patients according to cancer stage, histological type, grading, and state of surgical margins.

	Stage		Histological Type				Grading				Surgical Margins		
	Tis	T1a	SCC	Sarcomatoid Carcinoma	Leiomyosarcoma	High-Grade Dysplasia	1	2	3	X	RO	R Close	R1
%	45%	55%	95%	1%	1%	3%	14.4%	20.2%	4.8%	60.6%	74%	16.4%	9.6%
N (=104)	47	57	99	1	1	3	15	21	5	63	77	17	10

**Table 2:** Percentages and numbers of relapses, second tumors, second tumor relapses, relapses within 5 years of follow-up and after 5 years.

	Total Relapses	Second Relapses	Second Tumor	Second Tumor Relapses	Relapses Within 5 Years	Relapses After 5 Years
%	10.6%	2.9%	10.6%	1.9%	6.7%	3.8%
N (=104)	11	3	11	2	7	4

**Table 3:** Percentage and number of surgical options for patients with first and second relapses and disease-related deaths. (OPHL2A: Open partial horizontal laryngectomy type IIA).

	Treatment of First Relapse			Treatment of Second Relapse		Disease-Related Deaths
	Cordectomy	Total Laryngectomy	Partial Laryngectomy (OPHL2A)	Cordectomy	Total Laryngectomy	
%	73%	9%	18%	9%	18%	9%
N (=11)	8	1	2	1	2	1

**Table 4:** Results of GIRBAS scale analysis and mean value of parameters.

Parameters	Grade of Alteration				
	Normal (%)	Slight (%)	Moderate (%)	Severe (%)	Mean Value
Grade (G)	13 (26.5%)	13 (26.5%)	18 (36.7%)	5 (10.3%)	1.31
Instability (I)	35 (71%)	12 (25%)	2 (4%)	3 (6.2%)	0.33
Roughness (R)	18 (36.7%)	15 (30.6%)	13 (26.5%)	3 (6.2%)	1.02
Breathiness (B)	13 (26.5%)	17 (34.7%)	16 (32.6%)	3 (6.2%)	1.18
Asthenia (A)	28 (57.2%)	15 (30.6%)	5 (10.3%)	1 (2%)	0.57
Strain (S)	32 (65.3%)	10 (20.4%)	6 (12.3%)	1 (2%)	0.51

**Table 5:** Differences in distribution of roughness and strain parameters in patients who underwent speech therapy and who did not.

	Parameters	Grade of Alteration			
		Normal	Slight	Moderate	Severe
Speech therapy	R	19.2%	34.6%	34.6%	11.5%
	S	46.2%	30.8%	19.2%	3.8%
No speech therapy	R	56.5%	26.1%	17.4%	0%
	S	87%	8.7%	4.3%	0%

## Data Availability

No new data were created or analyzed in this study. Data sharing is not applicable to this article.
